# Plants and Fungal Products with Activity Against Tuberculosis

**DOI:** 10.1100/tsw.2005.80

**Published:** 2005-08-15

**Authors:** Marcus Vinicius Nora De Souza

**Affiliations:** FioCruz-Fundação Oswaldo Cruz, Instituto de Tecnologia em Fármacos-Far Manguinhos, Rua Sizenando Nabuco, 100, Manguinhos, 21041-25650, Rio de Janeiro, RJ, Brazil

**Keywords:** natural products, tuberculosis, antimycobacterial activity

## Abstract

Tuberculosis (TB) is becoming an ever more serious worldwide problem. This contagious disease kills four people every minute somewhere in the world and accounts for more than 2 million deaths per year. Due to the rapid spread of TB strains resistant to all the major anti-TB drugs on the market, and the association of TB with human immunodeficiency virus (HIV) infection in AIDS, we urgently need to develop new drugs to fight against TB. In this context, due to the importance of nature in the development of new drugs, the aim of the present review is to highlight a series of new and promising anti-TB agents derived from plants and fungi discovered between 2001 and 2005.

## INTRODUCTION

Nature is an incredible source of both simple and complex compounds that play an important role in the treatment of human diseases. For example, in the field of cancer and infectious disease, about 65% of the drugs on the market are of natural origin[1]. The importance of natural products in drug discovery can be noted in the large number of drugs derived from living organisms (including plants, animals, and insects), available against different classes of disease. As examples, one can mention the antibiotic penicillin G produced by the fungus *Penicillium chrysogenum*; the narcotic analgesic morphine extracted from the opium poppy *Papaver Somniferum*; the antimalarial quinine and artemisinin found in *Cinchona officinalis* and *Artemisia annua L.*, respectively; the anticancer vincristine and paclitaxel extracted from *Catharanthus roseus* and *Taxus brevifolia* Nutt, respectively; and the antiasthma ephedrine, isolated from the Ma huang herb (Fig. 1). Due to the importance of drug development from natural products today, much attention is being paid to the discovery of new molecules from nature with unexploited modes of action. In this context, the aim of the present article is to highlight new antituberculosis agents derived from plants and fungi discovered between 2001 and 2005.

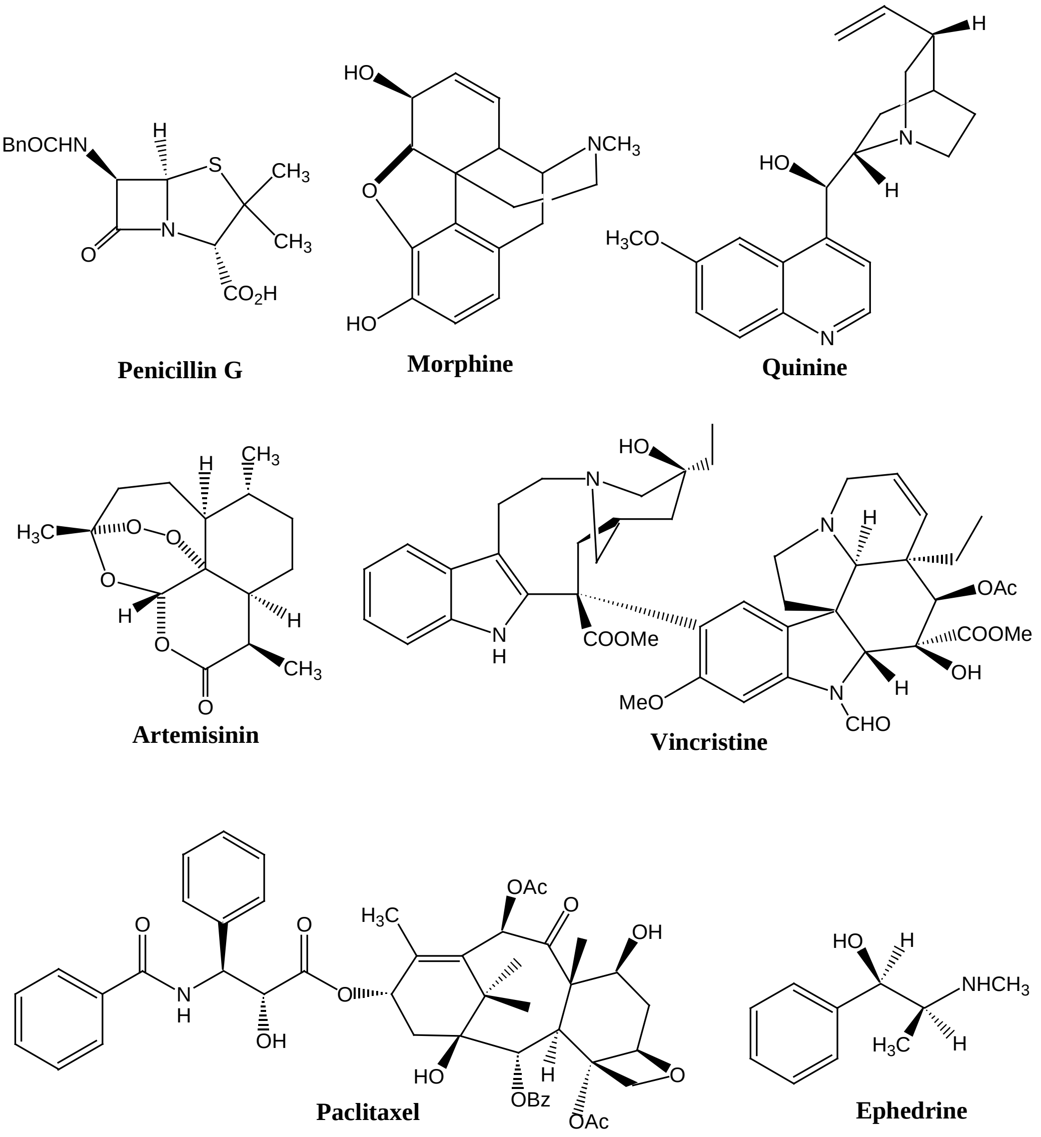

**FIGURE 1**

## TUBERCULOSIS

Tuberculosis (TB) is defined as a disease caused by the bacterium *Mycobacterium tuberculosis*, transmitted through the air that mainly affects and damages the lungs. It may, however, spread to any other organ system[2]. The resurgence of TB became a serious worldwide problem during the period from 1985–1992, particularly in people infected with the HIV virus. There are also other problems that contribute to the increasing incidence of TB, such as immigration, war, famine, homelessness, the lack of new drugs, and multidrug-resistant tuberculosis (MDR TB) that arise from inconsistent or partial treatment. Due to TB problems, the World Health Organization declared this disease a global health emergency in 1993. Today, TB with AIDS and malaria are the top three infectious disease killers in the world with, respectively, 2, 3, and 1 million people dying each year. Each year, 1% of the global population is infected and 5–10% of those infected become sick or infectious. The populations infected at present are: Africa (35%), Americas (18%), Eastern Mediterranean (29%), Europe (15%), Southeast Asia

(44%), and Western Pacific (35%). According to the statistics, without a coordinated control effort, TB will infect an estimated 1 billion more people by 2020, killing 70 million[3].

The first drug used in TB treatment appeared in 1946, 64 years after the discovery in 1882 by R. Koch of *M. tuberculosis,* with the introduction of the antibiotic streptomycin (Fig. 2). Following streptomycin, *p*-aminosalicylic acid (1949), isoniazid (1952), pyrazinamide (1954), cycloserine (1955), ethambutol (1962), and rifampin (rifampicin; 1963) were introduced as anti-TB agents. At present, the accepted treatment of TB involves a combination of the first-line drugs — isoniazid, pyrizinamide, ethambutol, and rifampicin (Fig. 3) — given over 6–9 months[4]. The combinations are very important to prevent the emergence of multiple drug-resistant organisms, which would lead to an ineffective treatment.

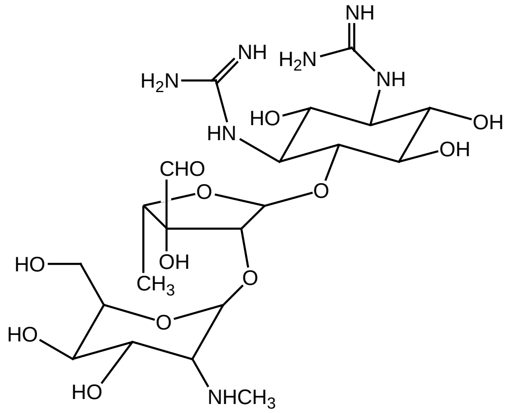

**Streptomycine**
**(1946)**

**FIGURE 2**

In spite of the worldwide problem caused by TB, it has been 30 years since a new drug was introduced to treat TB. Accordingly, the development of new drugs to fight against TB is urgently needed. In this context, nature provides a powerful source of new anti-TB compounds[5] that will be covered in this review, including promising natural products from plants and fungi discovered between 2001 and 2005.

## Natural Products from Plants

An important anti-TB activity was found by Xu and coworkers in the anti-HIV natural6product (+)-calanolide A (Fig. 4). This coumarin isolated from *Calophyllum lanigerum* var. *austrocoriaceum* (Guttiferae) was active *in vitro* against different strains of *M. tuberculosis*, including some resistant to the standard anti-TB drugs. Due to this important result, Xu and his research group screened other compounds related to pyranocoumarin that also demonstrated anti-TB activity[6]. These natural products are one of the first classes of reported natural compounds to possess important antimycobacterial and antiretroviral activities. Additionally, it is important to mention other coumarins that have been isolated and evaluated against *M. tuberculosis*[7].

Farnsworth and coworkers were the first to report antimycobacterial activity related to naphthopyrone compounds[8]. Quinquangulin and rubrofusarin (Fig. 5) extracted from stem and fruits of *Senna oblique*, a Peruvian plant, both had minimum inhibitory concentration (MIC) of 12 µg/mL against *M. tuberculosis*.

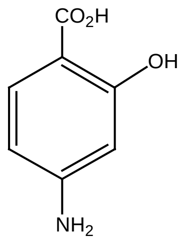

**p-aminosalicylic acid**
**1949**

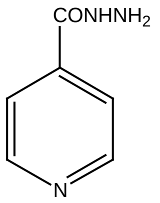

**Isoniazid**
**1952**

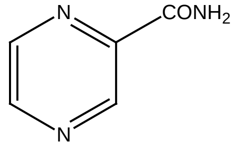

**Pyrazinamide**
**1954**

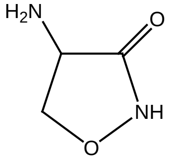

**Cicloserina**
**1955**

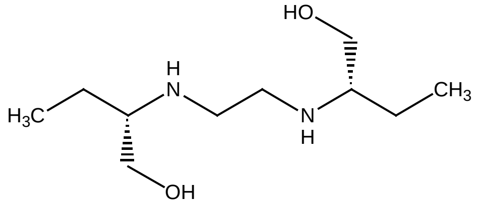

**Ethambutol**
**1962**

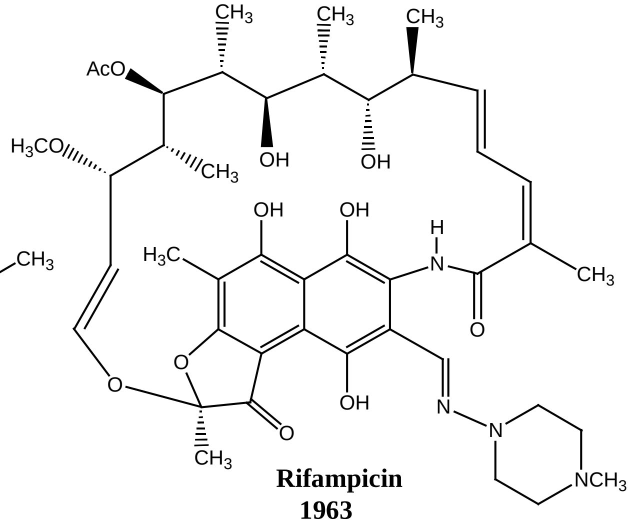

**Rifampicin**
**1963**

**FIGURE 3**

Chantrapromma and coworkers have isolated from the fruits of *Sapium indicum* L., a mangrove plant belonging to the family Euphorbiceae, three new phorbol esters **1**, **2,** and **8** together with another six known compounds **3**–**5** and **7**–**9**[9] (Fig. 6). This plant is widely present in the coastal areas of countries of Southeast Asia bordering the Indian Ocean and different phytochemical studies have resulted in the isolation of several phorbol esters. Based on their chemical studies of Thai medicinal plants, Chantrapromma and coworkers have elucidated three new compounds and evaluated the six phorbol esters for activity against *M. tuberculosis* H37Ra (Table 1). The MIC showed antimycobacterial activity between 3.12–200 µg/mL, however no activity was found for compounds **4** and **6** (MIC >200 µg/mL). The best result was found for compound **3** and it is interesting to observe that $4\beta$-isomers showed higher potency than $4\alpha$-isomers.

Four new compounds have been isolated from *Dendrolobium lanceolatum* by Kanokmedhakul and coworkers[10] (Fig. 7). This plant is a shrub 1–3 m in height and known in Thailand as “Kraduk-Khiat” or “Kraduk-Ueng”, where it is present in the northeastern part. In Thai medicine, this plant is used traditionally as a diuretic and for urinary disease. These four new compounds **10**–**13** have been tested against *M. tuberculosis* H37Ra with MIC values of 6.3, 12.5, 25, and 25 µg/mL, respectively.

In the search for the new bioactive constituents from Thai plants, Kanokmedhakul and coworkers have also isolated new diterpenes **14**, **17**–**19** from *Casearia grewiifolia* Vent. (Flacourtiaceae) commonly known in Thailand as “Kruai pa” or “Pha sam”[11] (Fig. 8). This plant is a shrubby tree 3–10 m in height, present in the northern and northeastern parts of Thailand, and decoctions of the bark and flowers are used traditionally as a tonic and a febrifuge, respectively. The four new terpenoids and the known terpenoids (**15** and **16**) were evaluated against *M. tuberculosis* and shown to possess promising antimycobacterial activities with MIC of 12.5 µg/mL for **14**–**16**, **18,** and **19**, and 25 µg/mL for **17**[11].

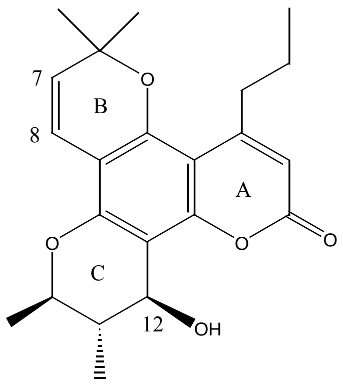

**(+) - Calanolide A**
Inhibition activity 96%
(MIC = 3.13 μ g $mL^{-1}$)

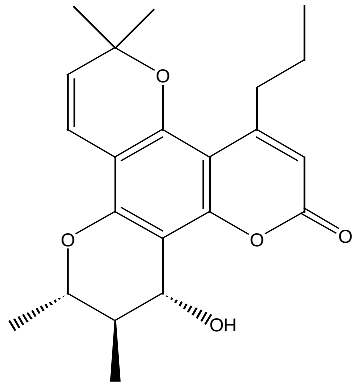

**(-) - Calanolide A**
Inhibition activity 98%
(MIC = 6.25 μ g $mL^{-1}$)

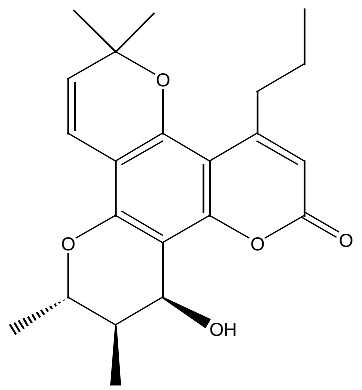

**(-) - Calanolide B**
Inhibition activity 99%
(MIC = 6.25 μ g $mL^{-1}$)

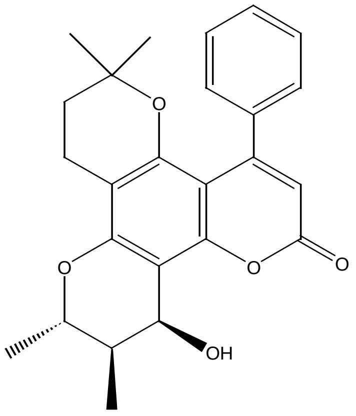

**(-) - 7,8-Dihydrocalanolide B**
Inhibition activity 98%
(MIC = 3.13 μ g $mL^{-1}$)

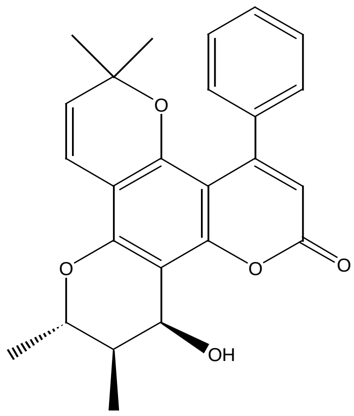

**Soulattrolide**
Inhibition activity 99%
(MIC = 3.13 μ g $mL^{-1}$)

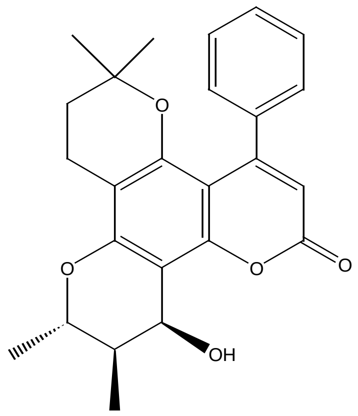

**7,8- Dihydrosoulattrolide**
Inhibition activity 99%
(MIC = 6.25 μ g $mL^{-1}$)

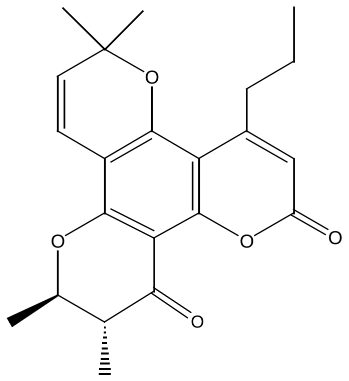

**(+) - 12-Oxocalanolide A**
Inhibition activity 78%
(MIC = >12.5 μ g $mL^{-1}$)

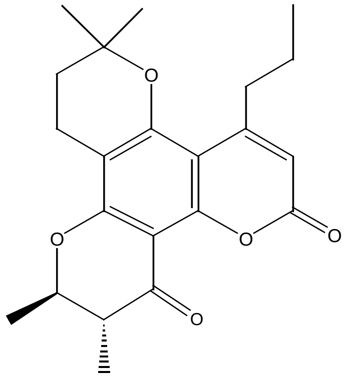

**(+/-) - 7,8 - Dihydro -12 - oxocalanolide A**
Inhibition activity 43%
(MIC = >12.5 μ g $mL^{-1}$)

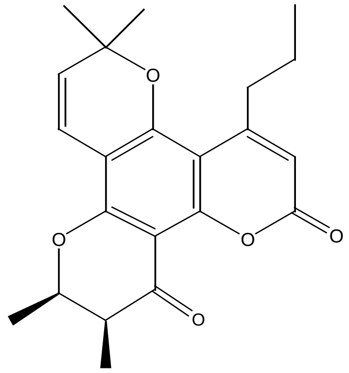

**(+/-) - Calanolide D**
Inhibition activity 57%
(MIC = >12.5 μ g $mL^{-1}$)

MIC - It is defined as a minimal inhibitory concentration of antibiotic that inhibits a bacterium.

**FIGURE 4**

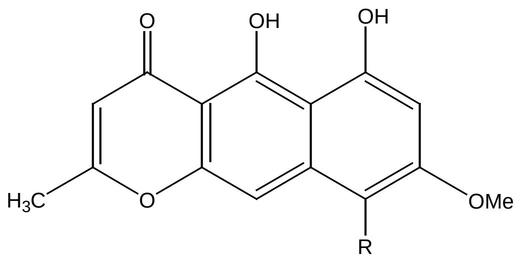

**R = $CH_3$ Quinquangulin**
**R = H Rubrofusarin**
Inhibition activity at 50 μg $mL^{-1}$ = 99%
(MIC = 12.5 μ g $mL^{-1}$)

**FIGURE 5**

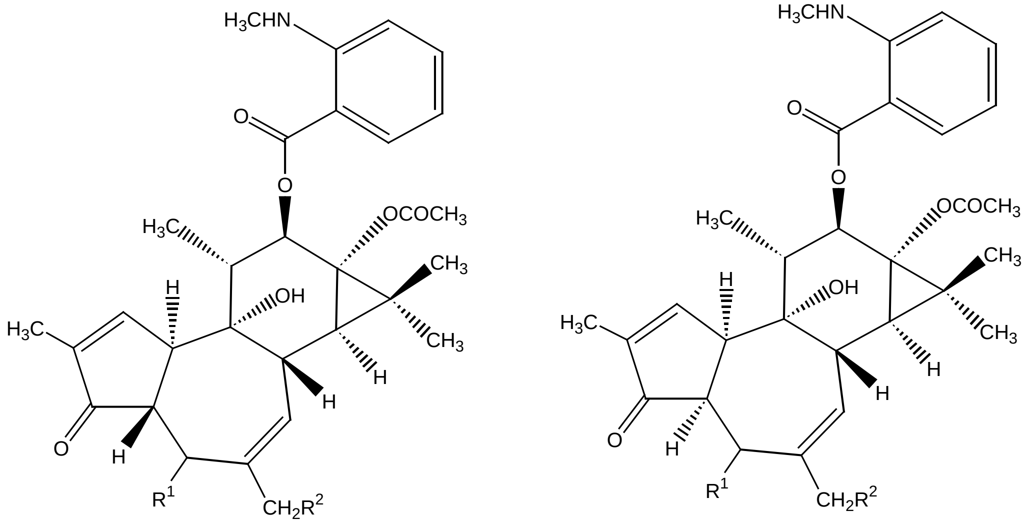

| | | | |
|---|---|---|---|
| **1** | $R^1$ = H, $R^2$ = H | **2** | $R^1$ = H, $R^2$ = H |
| **3** | $R^1$ = H, $R^2$ = OH | **4** | $R^1$ = H, $R^2$ = OH |
| **5** | $R^1$ = β–OH, $R^2$ = H | **6** | $R^1$ = β–OH, $R^2$ = H |
| **7** | $R^1$ = β–OH, $R^2$ = OH | **8** | $R^1$ = β–OH, $R^2$ = OH |
| **9** | $R^1$ = H, $R^2$ = CHO | | |

**FIGURE 6**

In an ethanolic extract of *Euclea natalensis* root bark, Lall and coworkers have identified two new compounds **21** and **22**, the known compound shinanolone **20** (Fig. 9), and other compounds[12]. This species occurs in different habitats, such as coastal and inland forests and bushveld. For example, it is widely present in tropical and subtropical Africa as well as on the east coast of South Africa, and it is important in African medicine. For example, the root bark is used by the Zulus to treat bronchitis, pleurisy, and chronic asthma[13]. Unfortunately, the two new compounds were not active against *M. tuberculosis*, however, shinanolone **20** showed activity against different Gram-positive bacterial strains and a drug-sensitive strain of *M. tuberculosis* (strain no. H37Rv ATCC 25923) at a concentration of 0.1 μg/mL[12].

**TABLE 1**
**The MIC of the Compounds**

| Compound | MIC (μg/mL) |
|---|---|
| 1 | 50 |
| 2 | 200 |
| 3 | 3.12 |
| 4 | >200 |
| 5 | 25 |
| 6 | >200 |
| 7 | 12.5 |
| 8 | 25 |
| 9 | 25 |
| Isoniazide | 0.04–0.09 |
| Kanamycin sulfate | 2.0–5.0 |

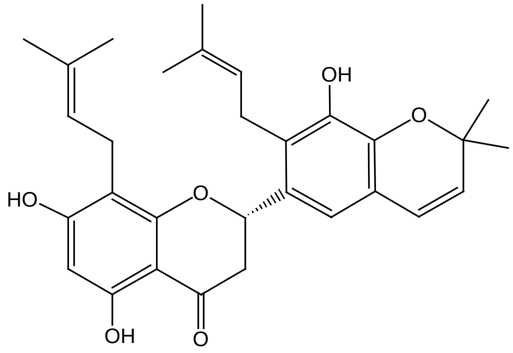

MIC = 6.3 μ g/ mL

**10**

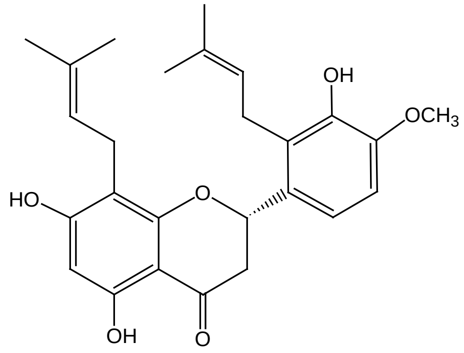

MIC = 12.5 μ g/ mL

**11**

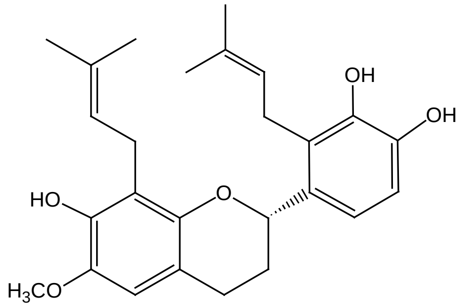

MIC = 25 μ g/ mL

**12**

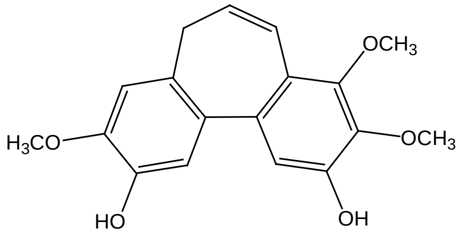

MIC = 25 μ g/ mL

**13**

**FIGURE 7**

Rukachaisirikul and coworkers have isolated a piperine dimer named chabamide **23** (Fig. 10) from stems of *Piper chaba* Hunter (piperaceae), which is a glabrous, fleshy plant cultivated in various parts of Thailand[14]. This dimer showed very good anti-TB activity with a MIC value of 12.5 μg/mL.

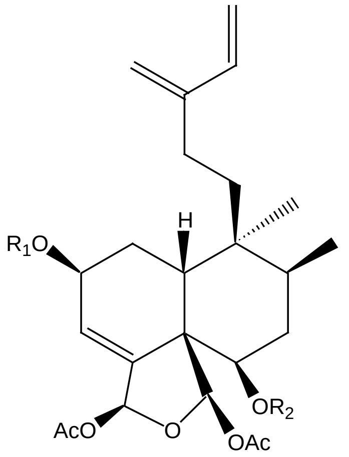

**14** $R_1 = (CH_3)_2CHCH_2CO$; $R_2 = CH_3$ MIC = 12.5 μ g / mL

**15** $R_2 = CH_3CH_2CH(CH_3)CO$; $R_2 = CH_3$ MIC = 12.5 μ g / mL

**16** $R_2 = CH_3CH_2CH(CH_3)CO$; $R_2 = H$ MIC = 12.5 μ g / mL

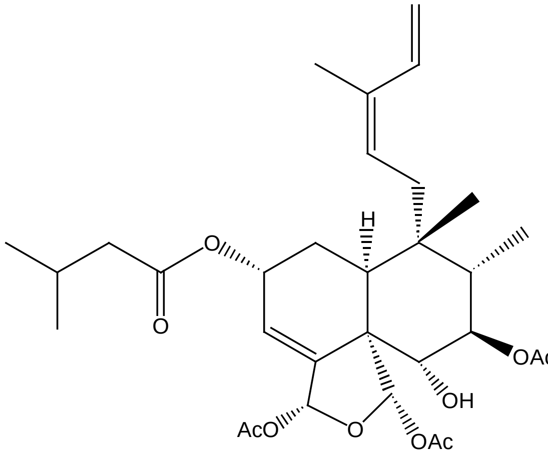

**17** MIC = 25 μ g / mL

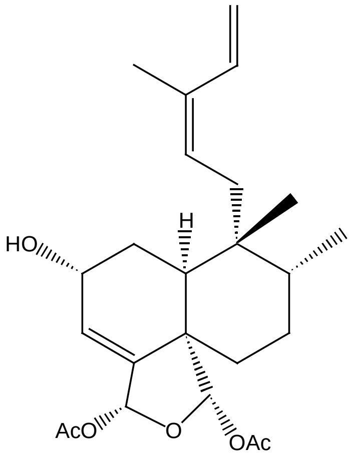

**18** MIC = 12.5 μ g / mL

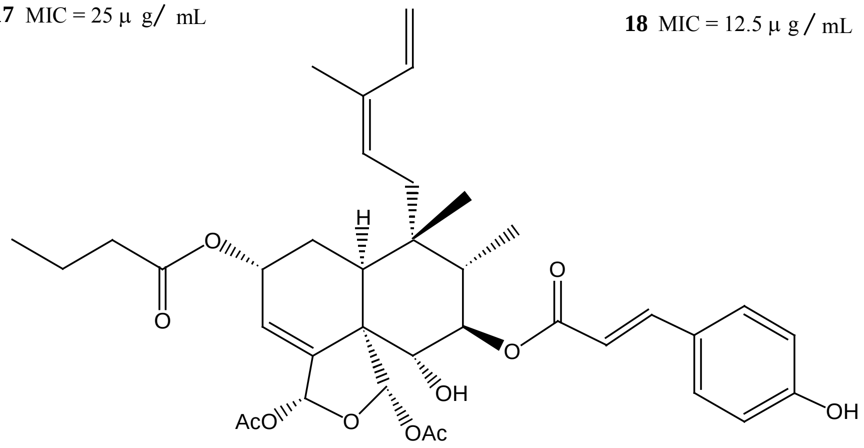

**19** MIC = 12.5 μ g / mL

**FIGURE 8**

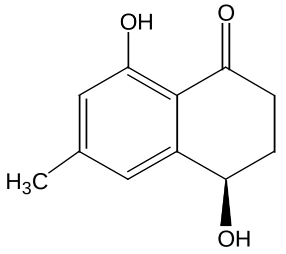

**Shinanolone**
MIC = 1.0 μg/ mL
**20**

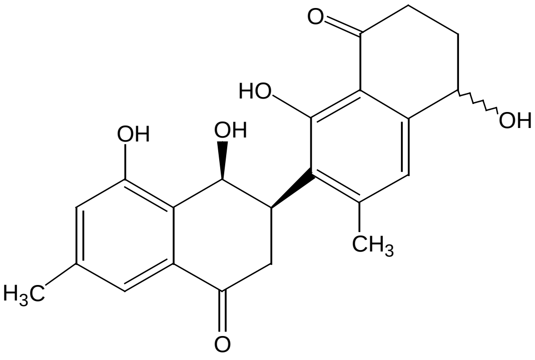

Inactive against *M. tuberculosis*

**21**

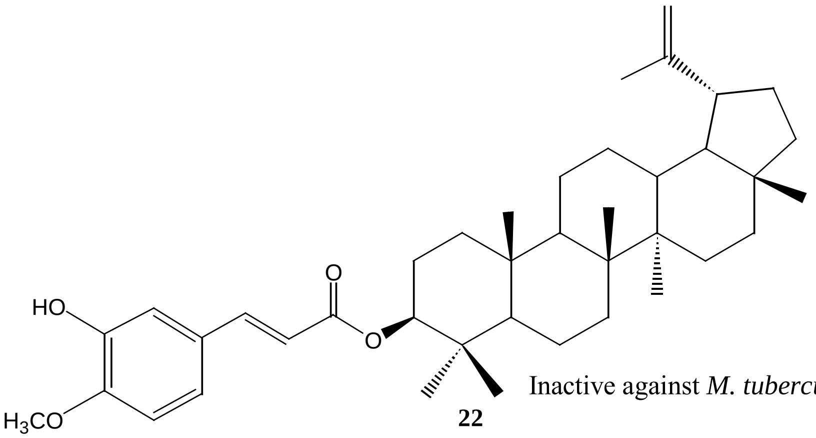

Inactive against *M. tuberculosis*

**22**

**FIGURE 9**

Fourteen compounds have also been isolated by Rukachaisirikul and coworkers from the fruits of the plant *P. sarmentosum*[15]. This species known in Thailand as "Cha-plu" is used as an expectorant. In Malay and Indonesia, the leaves and roots of this plant are used as treatment for different problems, such as asthma, toothache, and fungoid dermatitis on the feet. Studies of several extracts of this plant have been reported with a wide range of activity, such as antimalarial activity, neuromuscular blocking activity, and hypoglycemic effect. The compounds isolated by Rukachaisirikul and coworkers were evaluated and five of them, **24–28** (Fig. 11), presented good activity against *M. tuberculosis* (Table 2)[15].

Bucar and coworkers have investigated the antimycobacterial activity of 15 crude extracts prepared from 7 Ethiopian medicinal plants used to treat various infectious diseases[16]. Unfortunately, none of the tested extracts exhibited activity with the exception of the acetone fraction obtained from the stem bark of *Combretum molle*, which was active against *M. tuberculosis* at concentrations higher than 1 μg/mL.

The species *P. sanctum* is very abundant in the south-central region of Mexico, where it is commonly known as "acuyo", 'hierba santa", and "hoja santa". Its leaves have been prepared as a tea and employed against stomach cramps, coughs, bronchitis, TB, asthma, and colds. Mata and coworkers have isolated and elucidated 14 new compounds from the leaves and stem. The most active compound was the piperolactam A (Fig. 12), which showed a MIC value of 8 μg/mL[17].

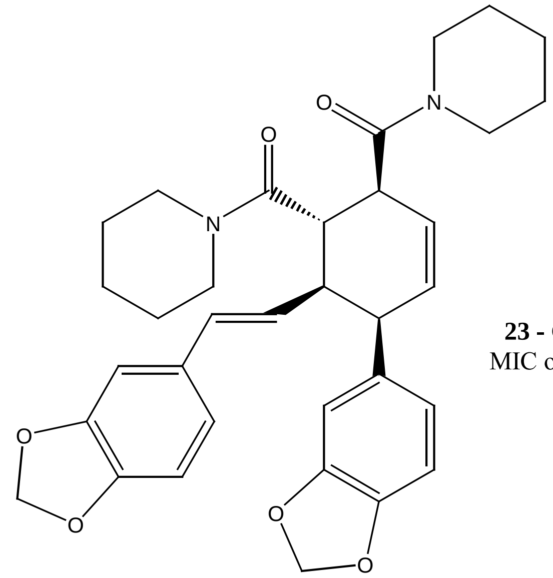

**23 - Chabamide**
MIC of 12.5 μg/mL

**FIGURE 10**

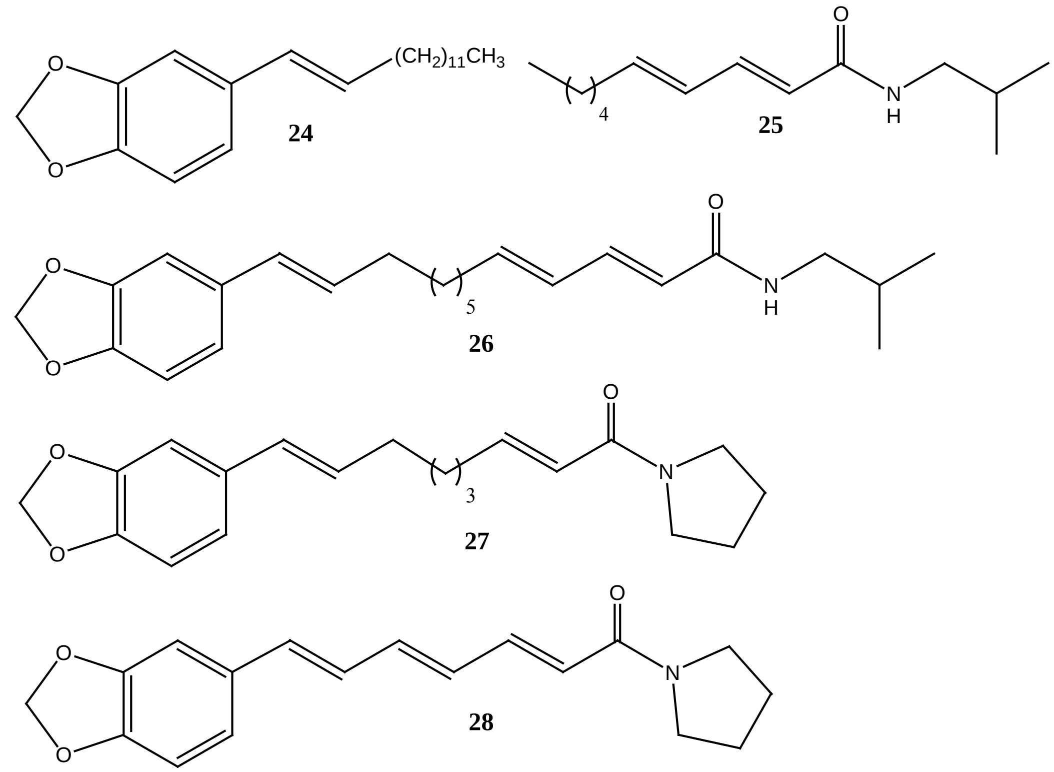

**FIGURE 11**

**TABLE 2**
**The MIC of the Compounds**

| Compound | MIC (μg/mL) |
|---|---|
| 24 | 25 |
| 25 | 25 |
| 26 | 50 |
| 27 | 50 |
| 28 | 50 |

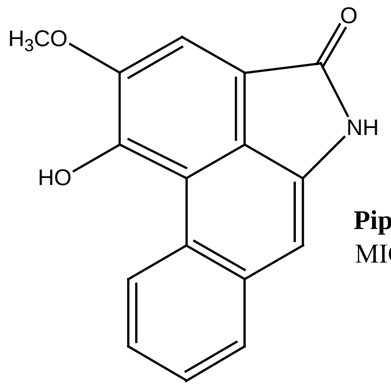

**Piperolactam A**
MIC - 8.0 μg/mL

**FIGURE 12**

Timmermann and coworkers have isolated and evaluated four new constituents of *Sapium haematospermum* against *M. tuberculosis*. This species is a plant native to drier regions of South America, commonly known in Argentina as "lecheryn", which is traditionally used in the treatment of teeth ailments[18]. The most active compound was the new pimarane, lachenerol A (Fig. 13), which showed a MIC value of 4 μg/mL. The presence of the hydroxyl group in C-19 in the pimarane, lachenerol B decreased the biological activity with a MIC of 128 μg/mL[18].

Timmermann et al. have also reported anti-TB activity of several sterol and terpenoids from Argentinean and Chilean plants[19]. For example, the Argentinean plants *Ruprenchtia triflora* Griseb. (Polygonaceae) and *Calceolaria pinnifolia* Cav. (Scrophulariaceae) were examined against *M. tuberculosis*[20]. The *R. triflora* is found in the semiarid Chaco region of South America and is used by indigenous peoples as a sedative. The $CH_2Cl_2$/MeOH extract furnished several sterols and a triterpene as the active components against *M. tuberculosis*, such as the unknown sterol **29** with a MIC value of 2.0 μg/mL (Fig. 14). The plant *C. pinnifolia* was also found in a semi-arid and mountainous region of northwestern Argentina, where it was used as an astringent. Among the ten diterpenes isolated from the diterpenes, **30** and **31** were the most active, each with a MIC of 4.0 μg/mL (Fig. 15).

The folk medicine in Thailand has commonly used the plant *Croton kongensis* for dysmenorrhea. Due to the importance of the genus *Croton* in Asian traditional medicine, Kittakoop and coworkers have isolated and evaluated two new 8,9-secokaurane diterpenes against *M. tuberculosis*, which possess MIC values of 25 and 6.25 μg/mL, respectively (Fig. 16)[21].

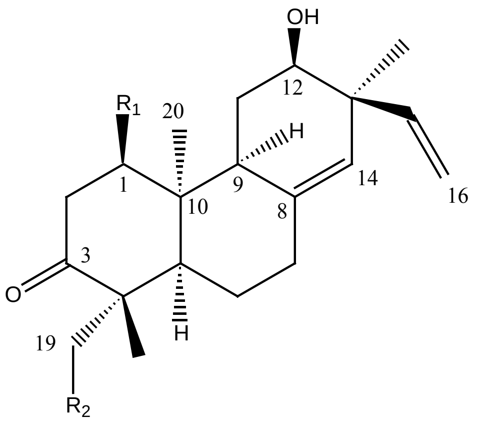

**Lachenerol A**

$R_1$ = H MIC - 4.0 µg/mL

**Lachenerol B**

$R_2$ = OH MIC - 128 µg/mL

**FIGURE 13**

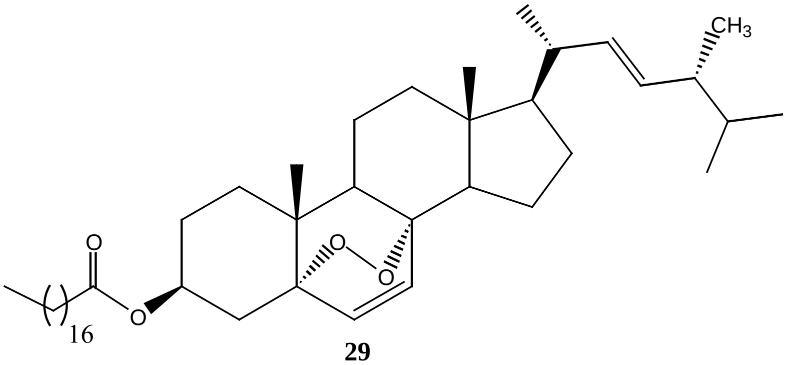

**29**

MIC - 2.0 µg/mL

**FIGURE 14**

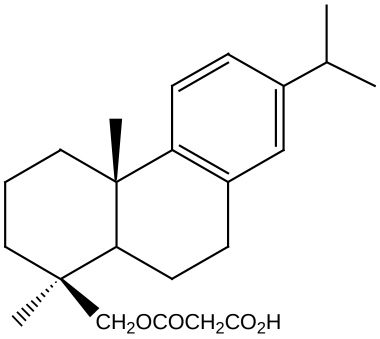

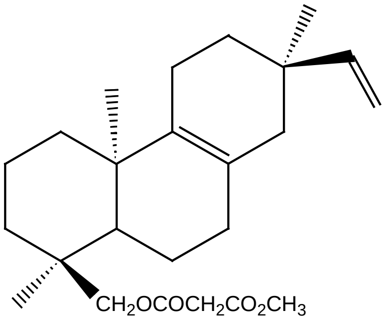

**30**
MIC - 4.0 µg/mL

**31**
MIC - 4.0 µg/mL

**FIGURE 15**

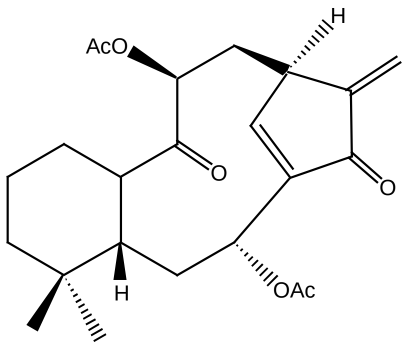

**32**
MIC - 25 μg/mL

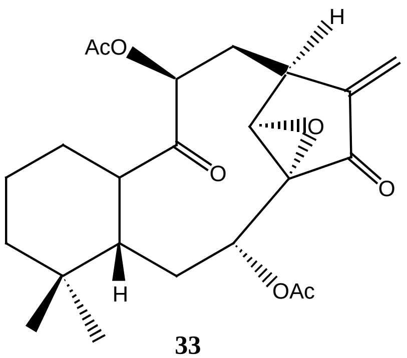

**33**
MIC - 6.25 μg/mL

**FIGURE 16**

Wright and co-workers have evaluated 43 plant species from 33 families *in vitro* against *M. tuberculosis*. This group has selected the plants on account of the traditional medicine for the treatment of TB and/or leprosy. The crude methanolic extracts from three of the plants (*Commiphora mukul*, *Psoralea corylifolia,* and *Sanguinaria canadensis)* were active against *M. aurum* and *M. bovis* BCG (MIC = 62.5 μg/mL)[22]. In these fractions, three known compounds have been isolated (Fig. 17) and evaluated (Table 3), the alkaloids sanguinarine and chelerythrine from the roots of *S. canadensis* and the known phenolic monoterpene, bakuchiol, from the seeds of *P. corylifolia*.

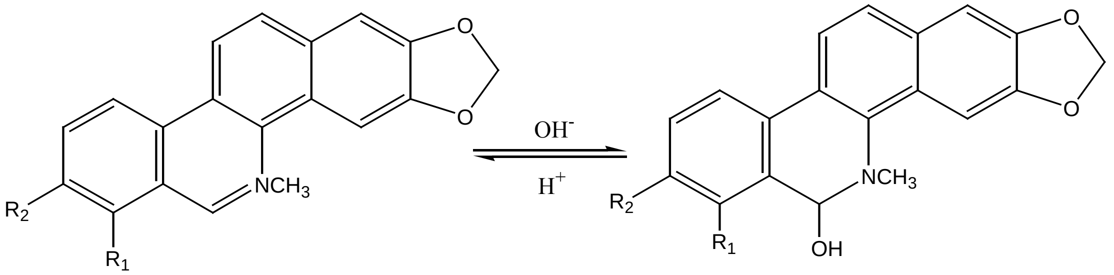

$R_1 + R_2 = OCH_2O$ **Sanguinarine**

$R_1 = R_2 = OCH_3$ **Chelerythrine**

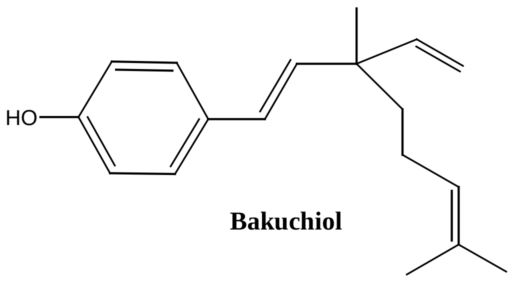

**Bakuchiol**

**FIGURE 17**

**TABLE 3**
**The MIC of the Compounds**

| **Compound** | **MIC (μg/mL)** | | |
|---|---|---|---|
| | ***M. aurum*** | ***M. smegmatis*** | ***M. bovis BCG*** |
| Sanguinarine | 25 | 112 | 67 |
| Chelerythrine | 19 | 76 | 37 |
| Bakuchiol | 62 | >1953 | 83 |

Karchesy and coworkers have isolated the new constituent (+)-totarol (Fig. 18) from *Chamaecyparis nootkatenses* outerbark, which was active against *M. tuberculosis* $H_{37}R_v$ with a MIC of 16 μg/mL[23]. This plant was collected from the Western slopes of the Olympic peninsula and it is used in traditional medicine by indigenous peoples in sweat baths for arthritis and rheumatism, infusions to sores, and swellings.

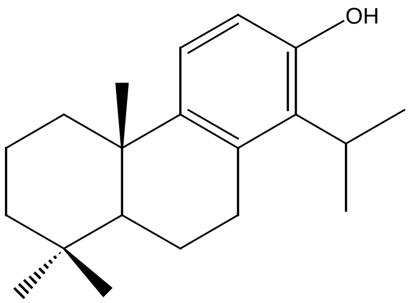

**(+) -Totarol**
MIC = 16 μg/mL

**FIGURE 18**

Licorine roots are largely used in traditional Chinese medicine in the treatment of different diseases, such as bronchial asthma, inflammation, and gastric and duodenal ulcers. Moller and coworkers have extracted and purified the licochalcone A (Fig. 19) from Chinese licorine roots, which was evaluated on 62 clinical isolates *Mycobacteria* sp.[24].

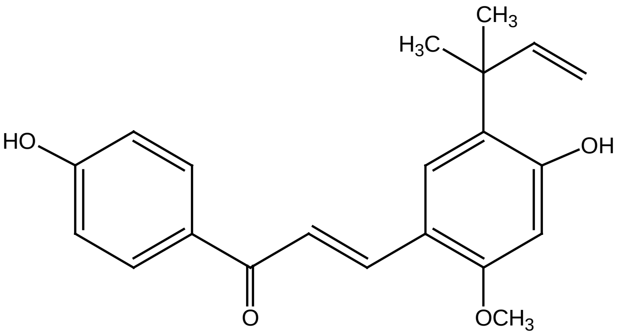

**Licochalcone A**

**FIGURE 19**

## Natural Products from Fungi

Kittakoop and coworkers have isolated and identified two pimarane diterpenes, diaporthein A and B (Fig. 20), from the culture broth of the fungus *Diaporthe* sp. BCC 6140[25]. These natural compounds have activity against *M. tuberculosis* Ra with a MIC of 200 and 3.1 µg/mL, respectively, using the drugs isonizid (MIC = 0.04–0.09 µg/mL) and kanamycin sulfate (MIC = 2.0–5.0 µg/mL) as references. The results have suggested that the carbonyl function in the C-7 position of diaporthein B (Fig. 12) is essential for the anti-TB activity[16].

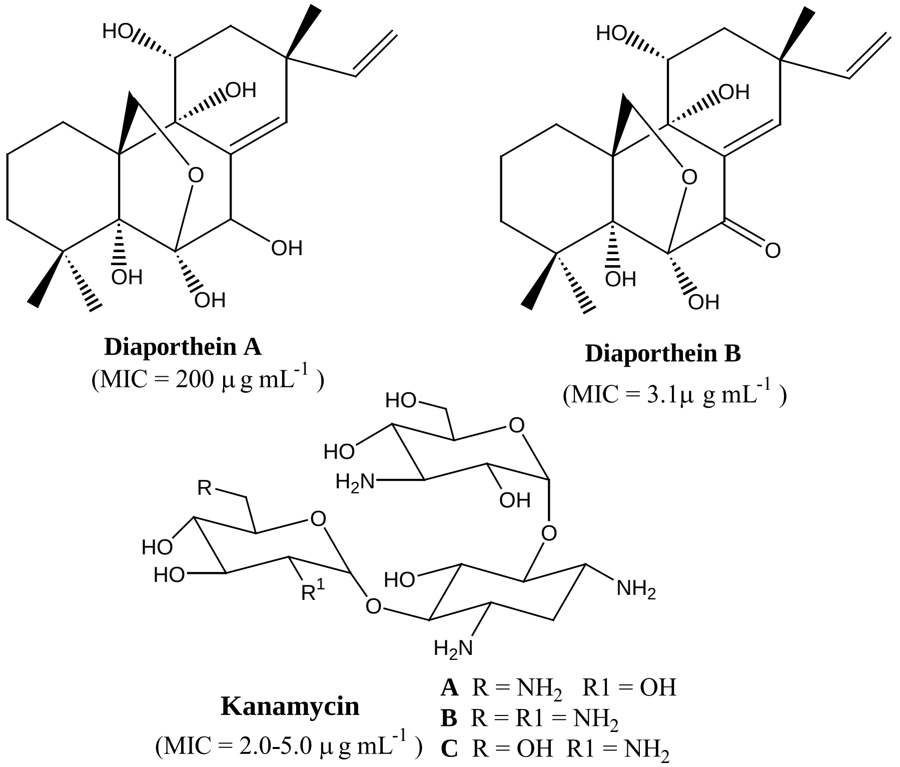

**FIGURE 20**

The new compounds, agonodepsides A and B (Fig. 21), were isolated and identified by Cao and coworkers from a nonsporulating, filamentous fungus (F7524) by using spectroscopic methods[26]. The inhibition of mycobacterial InhA enzyme, a 2-*trans*-enoyl-acyl-reductase involved in mycolic acid biosynthesis, which is a major lipid of the mycobacterial envelope, has demonstrated that agonodepside A had moderate activity, with an $IC_{50}$ value of 75 µ*M*, although agonodepside B (Fig. 21) was not active at 100 µ*M*.

The natural product hirsutellide A (Fig. 22) was isolated and elucidated by Kittakoop and coworkers[27] from the entomopathogenic fungus *Hirsutella kobayasii* BCC 1660. The antimycobacterial activity was assessed against *M. tuberculosis* H37Ra using the Microplate Alamar Blue Assay (MABA) with standard drugs, isoniazid and kanamycin, and this natural product showed a MIC with 6–12 µg/mL with no cytotoxic effect toward Vero cells at 50 µg/mL.

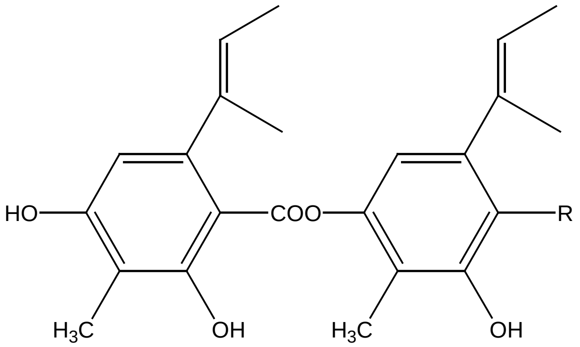

R = H; Agonodepside **A** $IC_{50}$ = 75μ M

R = $CO_2H$; Agonodepside **B** $IC_{50}$ = inativo at 100μ M

**FIGURE 21**

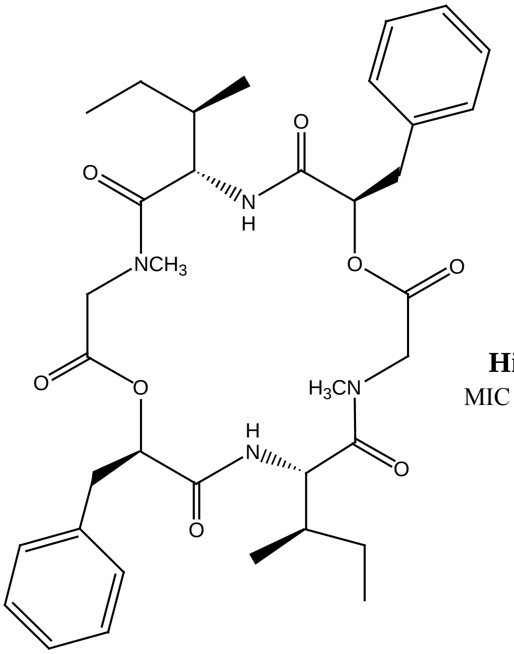

**Hirsutellide A**
MIC with 6-12 mg/mL

**FIGURE 22**

Mycothiol (Fig. 23) is a major, low molecular weight thiol found in most actinomycetes. This compound was first isolated as the disulfide from *Streptomyces* sp. AJ9463 by Sakuda and coworkers and it is responsible for protecting the organism from oxidative stress and removes electrophilic, alkylating agents and other toxins[28]. Mycothiol plays a role similar to that of glutathione in eukaryotes and Gram-negative bacteria. Due to their importance, mycothiol is an important target for the design of new molecules against TB.

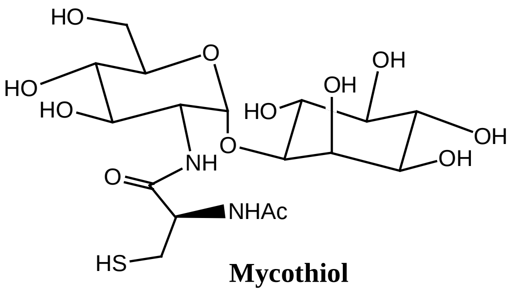

**FIGURE 23**

Bewley and coworkers have studied the identification of inhibitors of a novel detoxification enzyme, mycothiol-*S*-conjugate amidase (MAC), which is unique to actinomycetes. This group has evaluated over 1,500 extracts derived from different natural sources, including 300 species from terrestrial fungal cultures, for their ability to inhibit MAC[29]. This study led to the identification of 13 natural products inhibitors, of which 10 of them derived from marine sources and 3 from terrestrial fungus gliotoxin, *S,S*-dimethyl gliotoxin, and physcion from *Mycena* sp. and *Aspergillus* sp., respectively (Fig. 24)[29].

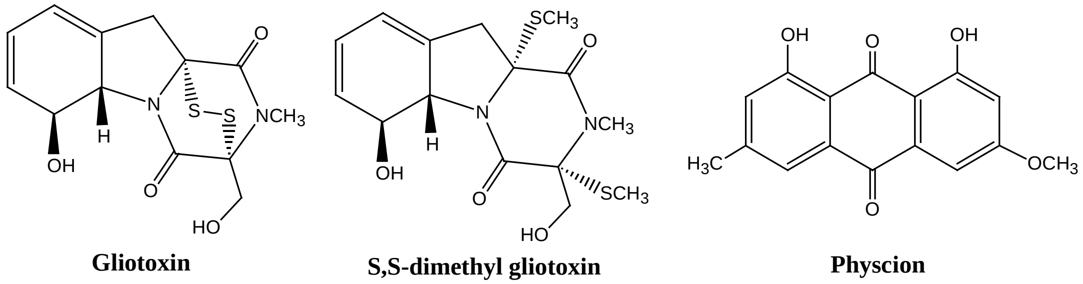

**FIGURE 24**

Kongsaeree and coworkers have isolated three novel dihydroisocoumarins produced by *Geotrichum* sp., an endophytic fungus isolated from *Crassocephalum crepidoides* S. Moore[30] (Fig. 25). Endophytic fungi are present in the intracellular spaces of vascular plants and are an important source of new, biologically active, secondary metabolites. The MIC values of the compounds **34**–**36** were, respectively, 25 μg/mL, 50 μg/mL, and inactive. That suggests that the double bond $C_{11}$-$C_{12}$ and the aliphatic group $C_{14}$-$C_{17}$ are important for the biological activity.

Kanokmedhakul and coworkers have isolated a novel anthraquinone-chromanone (named chatomanone) together with other known compounds from the fungus *Chaetomium globosum* KMITL-N802 (Fig. 26). This compound was evaluated *in vitro* against *M. tuberculosis* with MIC value of 216 μ*M*[31].

The evaluation of antimycobacterial activity of the lichenicolous fungus *Microsphaeropis* sp. BCC 3050 was made by Isaka and coworkers. This fungus was isolated from a lichen sample, *Dirinaria applanata,* collected from Phu Tee-Suan-Sai forest in Loei province, Northeastern Thailand. In this fungus, a new preussomerin **37** (Fig. 27) was isolated with others compounds, which showed a MIC value of 3.12 μg/mL[32].

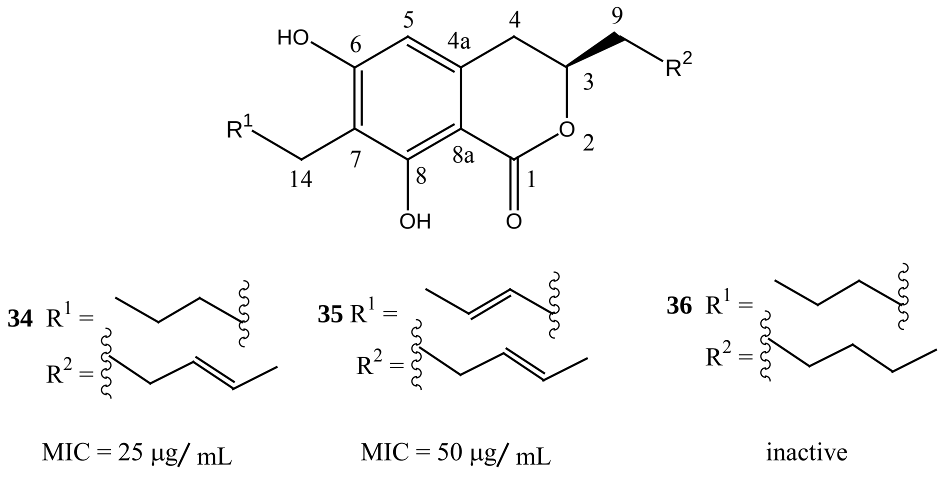

**FIGURE 25**

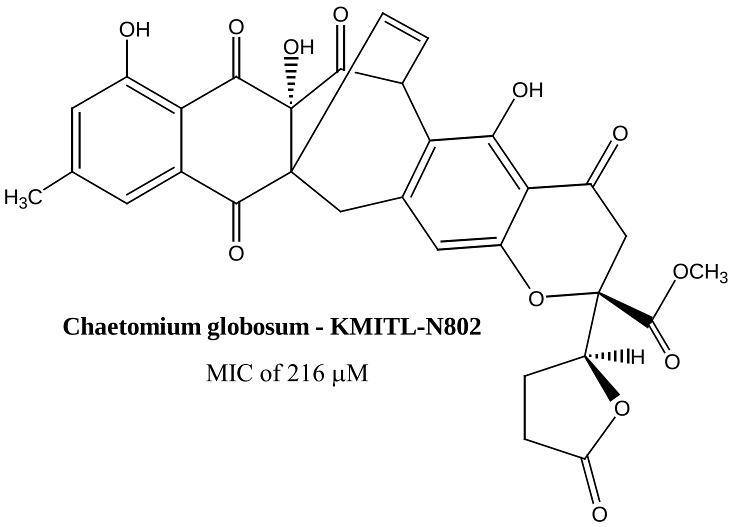

**FIGURE 26**

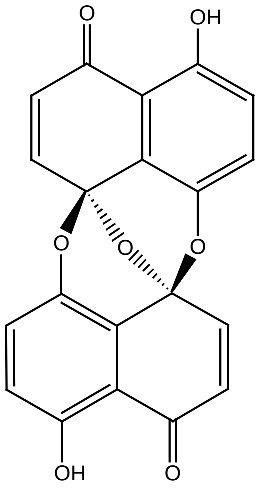

**37**
MIC = 3.12 μg/ mL

**FIGURE 27**

## CONCLUSION

To combat the rapid spread of multidrug-resistant TB strains effectively, it is imperative to have new drugs and strategies against this disease. Natural products, particularly from plants and fungi, offer unique chemical diversity with the potential to open a new era in the treatment of TB.
